# Reduced Levels of Selenium and Thioredoxin Reductase in the Thoracic Aorta Could Contribute to Aneurysm Formation in Patients with Marfan Syndrome

**DOI:** 10.3390/ijms241310429

**Published:** 2023-06-21

**Authors:** María Elena Soto, Israel Pérez-Torres, Linaloe Manzano-Pech, Elizabeth Soria-Castro, Almilcar Morales-Marín, Edgar Samuel Ramírez-Marroquín, Humberto Martínez-Hernández, Valentín Herrera-Alarcón, Verónica Guarner-Lans

**Affiliations:** 1 Department of Immunology, Instituto Nacional de Cardiología Ignacio Chávez, Juan Badiano 1, Sección XVI, Tlalpan, México City 14080, Mexico; mesoto50@hotmail.com; 2 Department of the Cardiovascular Line, Division of the American British Cowdray Medical Center, Sur 136 No. 116 Col. Las Américas, México City 01120, México; 3 Department of Cardiovascular Biomedicine, Instituto Nacional de Cardiología Ignacio Chávez, México City 14080, Mexico or israel.perez@cardiologia.org.mx (I.P.-T.); loe_mana@hotmail.com (L.M.-P.);; 4 Deparment Cardiothoracic Surgery Instituto Nacional de Cardiología Ignacio Chávez, México City 14080, Mexico; amilcar_mm@live.com (A.M.-M.);; 5 Department of Physiology, Instituto Nacional de Cardiología Ignacio Chávez, Juan Badiano 1, Sección XVI, Tlalpan, México City 14080, Mexico

**Keywords:** marfan syndrome, selenium, thoracic aneurysm, thioredoxin, oxidative stress

## Abstract

Marfan syndrome (MFS) is an autosomal dominant disorder caused by a heterozygous mutation of the FBN1 gene. MFS patients present oxidative stress that disturbs redox homeostasis. Redox homeostasis depends in part on the enzymatic antioxidant system, which includes thioredoxin reductase (TrxR) and glutathione peroxidases (GPx), both of which require an adequate concentration of selenium (Se). Therefore, the aim of this study was to determine if Se levels are decreased in the TAA of patients with MFS since this could contribute to the formation of an aneurysm in these patients. The results show that interleukins IL-1β, IL-6 TGF-β1, and TNF-α (*p* ≤ 0.03), and carbonylation (*p* ≤ 0.03) were increased in the TAA of patients with MFS in comparison with control subjects, while Se, thiols (*p* = 0.02), TrxR, and GPx (*p* ≤ 0.001) were decreased. TLR4 and NOX1 (*p* ≤ 0.03), MMP9 and MMP2 (*p* = 0.04) and NOS2 (*p* < 0.001) were also increased. Therefore, Se concentrations are decreased in the TAA of MFS, which can contribute to a decrease in the activities of TrxR and GPx, and thiol groups. A decrease in the activities of these enzymes can lead to the loss of redox homeostasis, which can, in turn, lead to an increase in the pro-inflammatory interleukins associated with the overexpression of MMP9 and MMP2.

## 1. Introduction

Marfan syndrome (MFS) is one of the most common hereditary collagen diseases. It is caused by a heterozygous mutation in the gene that encodes FBN1, which is located in chromosome 15q21.1. This mutation has an approximate incidence of 1–3 per 5000 people, affects both sexes and is distributed worldwide [1]. MFS is an autosomal dominant disorder with pleiotropic features that include skeletal abnormalities, ectopia lentis, and dilatation of aortic aneurysms. The main clinical problem is thoracic aortic aneurysm (TAA) with a risk of dissection and with a higher risk of aortic rupture when the root diameter approaches 5 cm. This feature is present in more than 80% of adults with MFS [2]. The diagnosis of MFS is based on clinical criteria and may be complemented by a genetic confirmation of the pathogenic variants in FBN1 as described in Ghent’s nosology [3].

The FBN1 gene codes for the protein fibrillin and is essential for the formation of elastic fibers (EF) in connective tissue [4]. FBN1 gene mutations may cause deregulation of the transforming growth factor beta-1 (TGF-β1) signaling pathway, which plays a very important role in maintaining the integrity of the extracellular matrix (ECM) [5]. The ECM gives mechanical strength and structure to the aorta and provides communication between endothelial cells to regulate their differentiation and the proliferation of vascular smooth muscle cells (VSMC) [6]. There is an increase in TGF-β1 in MFS patients that could favor the transcription of metalloproteinases (MMPs) such as MMP9 and MMP2 that participate in a high proliferation and migration of VSMC with degradation of the ECM. The excess TGF-β1 may also change the functional activity of the fibrillin protein, which leads to a decrease in the EF in a positive feedback process and causes increased growth and destabilization of the ECM [7]. In this sense, histological observations of the aortic aneurysm wall in MFS show degeneration of the media zone of the aorta with the disappearance of VSMC, disorganization, breakage of EF, an excess of collagen fibers, accumulation of mucopolysaccharides, and cystic necrosis [8]. These alterations allow progressive aortic dilatation with possible breakage, fatal consequences, and premature death [9].

MFS patients also present oxidative stress (OS) associated with a deficiency in the enzymatic and non-enzymatic antioxidant systems, including a decrease in the enzymes that employ glutathione (GSH), such as glutathione peroxidase (GPx), glutathione reductase, and glutathione-S-transferase [9]. A decrease in the expression or activities of these enzymes can contribute to chronic inflammation and the loss of redox homeostasis, and it is associated with an increase and a decrease in the inducible nitric oxide synthase (NOS2) and endothelial nitric oxide synthase (NOS3), respectively [10]. Redox homeostasis equilibrium depends on the enzymatic and non-enzymatic antioxidant systems, and it is essential for the normal functionality of the arterial wall due to its protective function against different pathologies that contribute to damage of the arterial wall and endothelium [11]. In this sense, some enzymes of the antioxidant system, such as the families of thioredoxin reductases (TrxR) and GPx, require an adequate concentration of selenium (Se) for the efficacy of their catalytic activity against reactive oxygen species (ROS) [12]. Se participates in the synthesis of approximately 25 selenoenzymes that have a selenocysteine group (also called the 21st amino acid). For the synthesis of this group, selenocysteine synthase is required in the catalytic site. This site results from the union of the amino acid cysteine with a Se atom that replaces the usual sulfur atom, resulting in a potential reduction against ROS. A deficiency in this essential micronutrient and numerous mutations in selenoenzymes lead to disorders [13] in the central nervous system, muscular mass, and cardiovascular system. It also affects the normal physiological state of bone and cartilage through oxidative stress and immune reactions and results in arthropathies such as Kashin–Beck disease, rheumatoid arthritis, osteoarthritis, osteoporosis, and Keshan disease [14]. Low Se levels have been demonstrated in the aorta and heart in animal models and humans [15]. Conversely, Se at high concentrations may lead to selenosis [16].

There is scant evidence of the participation of Se in MFS. However, recently, low Se concentrations have been reported in the thoracic aortic aneurysm (TAA) of patients with Loeys–Dietz syndrome that contribute to the development of the thoracic aneurysm and oxidative background [10]. This syndrome is a more aggressive variant of the MFS with mutations in the TGF-*β*R1, TGF-*β*R2, and SMAD3 genes. Therefore, the aim of this study was to determine if Se levels are decreased in the TAA of patients with MFS since this could contribute to the formation of an aneurysm in these patients.

## 2. Results

### 2.1. Demographic Characteristics of the MFS Patients

A total of 19 patients were included, all of whom fulfilled more than two Ghent criteria—2 (13%) with two, 11 (54%) with three, and 6 (33%) with four criteria. As for the systemic score criterion, in which a score of at least 7 points out of a total of 20 is required, the total score for each patient is shown in Table 1. The Ghent criterion for each patient is also shown. The frequencies of each of the items included in the systemic score were: a positive family history of MS 13 (54%), facial signs 23 (96%), ectopia lentis 11 (46%), pectus excavatum 17 (71%), pectus carinatum 5 (21%), Walker–Murdoch sign 23 (96%), Steinberg sign 24 (100%), flat feet 21 (88%), dural ectasia 19 (79%), stroke > height 22 (92%), scoliosis 21 (88%), striae 19 (79%), myopia 21 (88%), mitral valve prolapse 14 (58%), and acetabular protrusion 3 (13%).

Table 2 shows the different general variables of MFS patients and control subjects (CS). These are demographic variables and serum biochemistry variables.

### 2.2. Inflammatory Interleukins, Se, and Carbonylation Levels

Table 3 shows that the concentrations of IL-6 and TGF-β1 were statistically increased in MFS patients vs. CS (*p* ≤ 0.03). The same trend was observed in the number of carbonyls per mg of protein (*p* = 0.03). However, Se concentrations and thiol groups (*p* = 0.02) in the MFS patients were decreased in comparison with the CS.

### 2.3. Enzymatic Activities and Expressions

**Figure 1:**
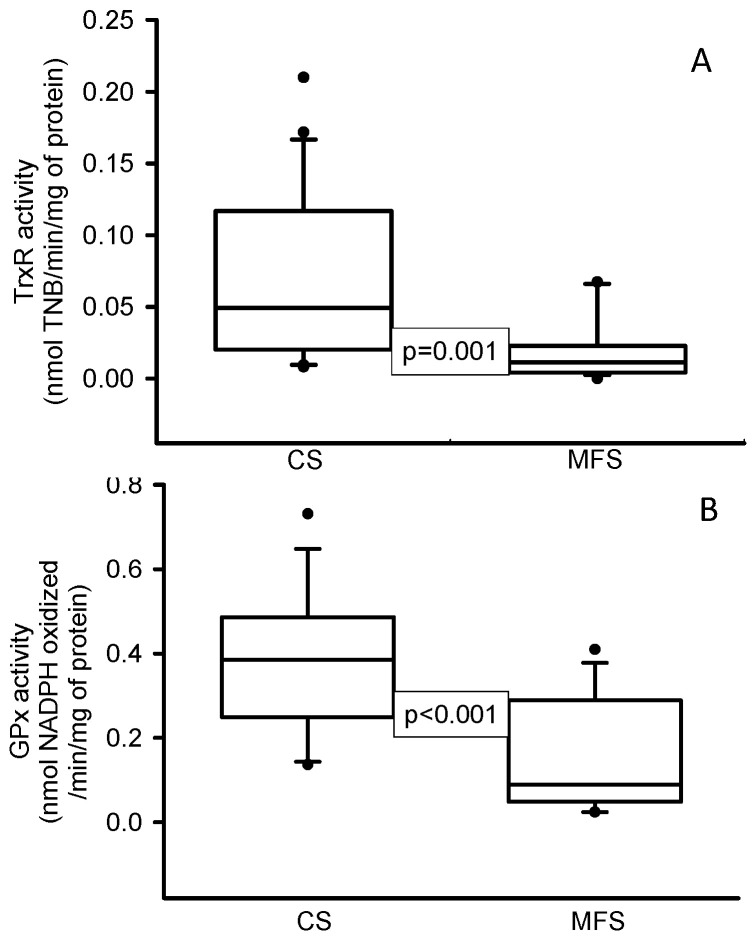
Enzymatic activities of TrxR (**A**) and GPx (**B**) in TAA from MFS patients in comparison with a segment of the thoracic aorta from CS. Values are expressed as the median and Min–Max range. Abbreviation: CS = control subjects, GPx = glutathione peroxidase, TrxR = thioredoxin reductase, MFS = Marfan syndrome.

**Figure 2:**
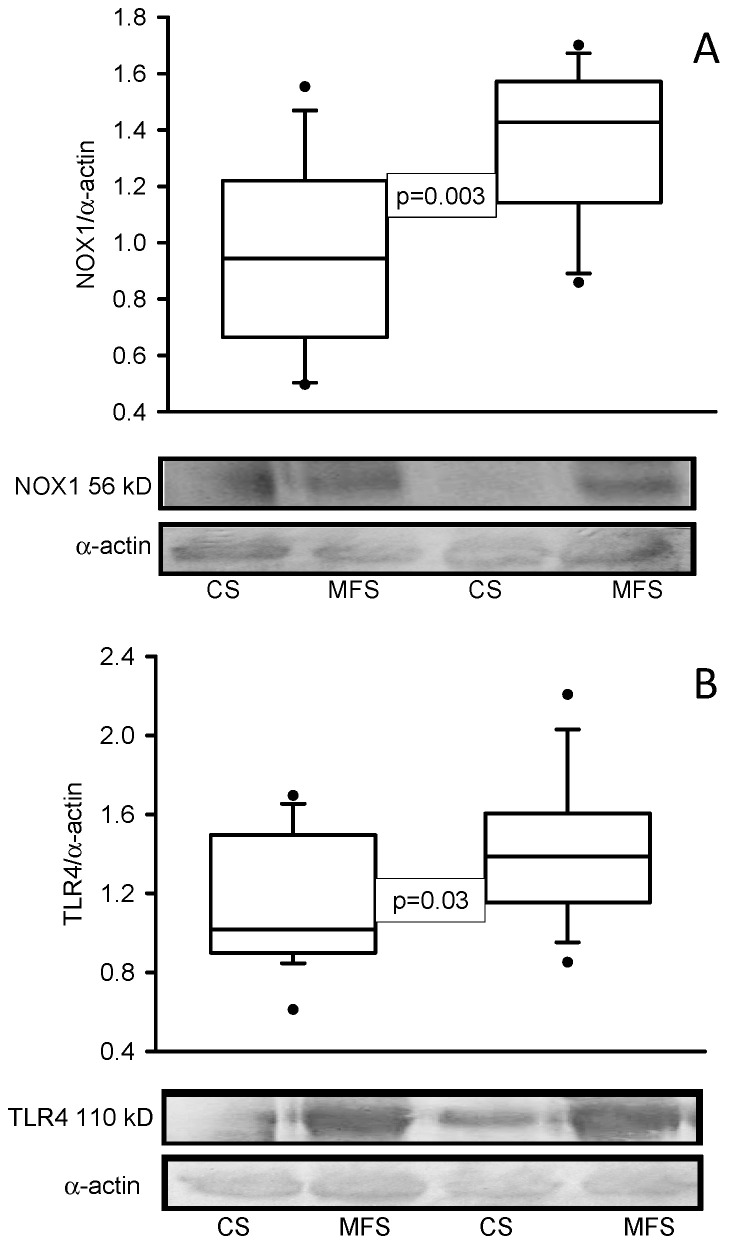
Protein expression of NOX1 (**A**) and TLR4 (**B**) in TAA from MFS patients in comparison with a segment of the thoracic aorta from CS. The Western blots are representative examples. Values are expressed as the median and Min–Max range. Abbreviation: MFS = Marfan syndrome, CS = control subjects.

The enzymatic activities of TrxR (*p* = 0.001, Figure 1A) and GPx (*p* < 0.001, Figure 1B) were decreased in the MFS patients in comparison with the CS. In addition, the expressions of NOX1 (*p* < 0.001, Figure 2A) and TLR4 (*p* = 0.01, Figure 2B) were increased in the TAA homogenate of the MFS patients in comparison with homogenates from a segment of the thoracic aorta from the CS.

### 2.4. Immunohistochemistry

**Figure 3:**
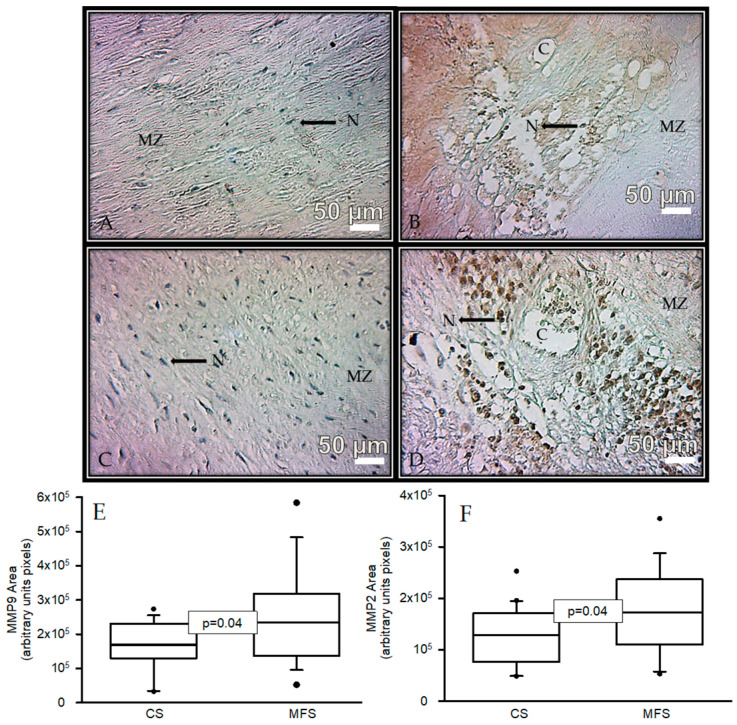
Representative microphotograph at 32× with immunostaining for an antibody against MMP9 (**A**,**B**) and immunostaining for an antibody against MMP2 (**C**,**D**) in the muscular zone in segments of thoracic aorta from CS and TAA from MFS patients, respectively. The graphics (**E**,**F**) represent the densitomorphometry of the analyzed area with immunostaining for antibodies against MMP9 and MMP2, respectively. The values are expressed in arbitrary units (pixels). The data are presented as median and Min–Max range. Abbreviations: C = cystic necrosis, CS = control subject, MSF = Marfan syndrome, MZ = muscular zone, N = cell nucleus.

Figure 3 shows the immunolabeling area (brown color) in the muscular zone when an antibody against MMP9 was used in a segment of the thoracic aorta from CS and a segment of the TAA from MFS patients (A and B, respectively). A statistically significant increase was present in the area of immunolabeling in MFS patients (*p* = 0.04, Figure 3E) when images were analyzed by densitomorphometry. The same tendency was observed in the immunolabeling area in the muscular zone when an antibody against MMP2 was used. There was a significant increase in the MFS patients in comparison with the CS (*p* = 0.04, Figure 3F).

**Figure 4:**
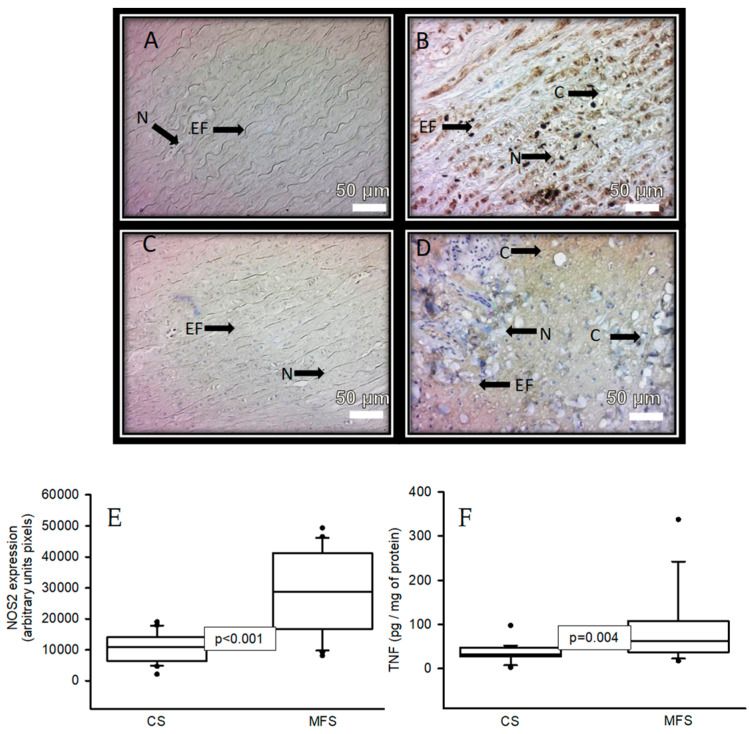
Representative microphotograph at 32× with immunostaining for an antibody against NOS2 (**A**,**B**) and an antibody against TNF (**C**,**D**) in the muscular zone in a segment of the thoracic aorta from CS vs. a segment from the TAA from MFS patients, respectively. The graphics (**E**,**F**) represent the densitomorphometry of the analyzed area with the immunostaining for antibodies against NOS2 and TNF, respectively. The values are expressed in arbitrary units (pixels). The data are presented as median and Min–Max range. Abbreviations: C = cystic necrosis, CS = control subject, EF = elastic fibers, MSF = Marfan syndrome, N = cell nucleus.

Figure 4 shows the immunolabeling area (brown color) in the muscular zone when an antibody against NOS2 was used in a segment of the thoracic aorta from CS and in a segment of the TAA from MFS patients (A and B, respectively). A statistically significant increase was present in the area of immunolabeling in MFS (*p* < 0.001, Figure 3E) when the images were analyzed by densitomorphometry. The same tendency was observed in the immunolabeling area in the muscular zone when an antibody against TNF-α was used. There was a significant increase in the MFS patients in comparison with the CS (*p* = 0.01, Figure 3F).

**Figure 5:**
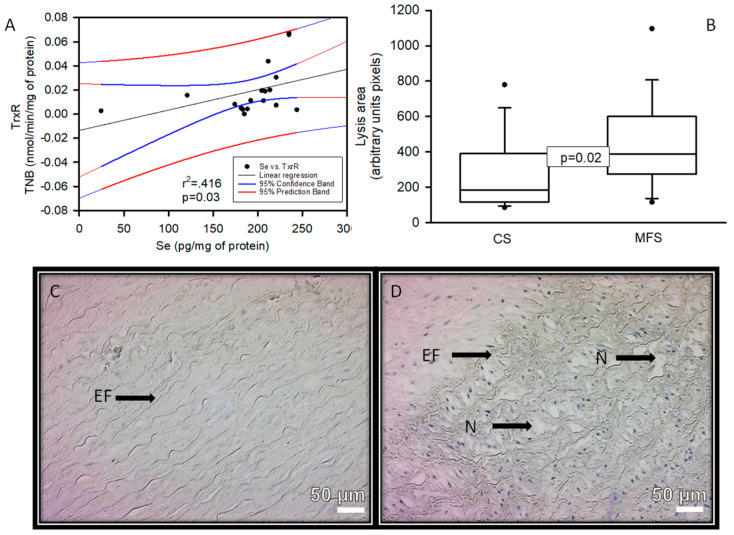
Linear regression of Se concentration vs. TrxR activity (**A**) in the TAA homogenate of the MFS patients, constant variance test Spearman rank correlation. Densitomorphometry of the area of lysis (cystic necrosis) in the zone media muscular (**B**) And representative microphotograph at 32× (**C**,**D**) in the TAA of the MFS patients vs. a segment of the thoracic aorta from CS, respectively. Cystic necrosis with rupture of elastic fibers is observed in MFS patients. Abbreviations: EF = elastic fibers, N = cystic necrosis.

There was a positive correlation with a significant difference (*p* = 0.03 and r^2^ = 0.416), between Se concentration and TrxR activity in the TAA homogenate of the MFS patients (Figure 5).

## 3. Discussion

There is scant evidence on the participation of Se in MFS patients, and, therefore, the objective of the current study was to determine if Se levels are decreased in the TAA of patients with MFS since this could contribute to the formation of an aneurysm in these patients. The essential antioxidant element Se is absorbed in the form of organic compounds and in the presence of vitamins A, D, and E. The Se is incorporated into selenoproteins by selenocysteine synthase, mostly in the liver but also in all other cells to a lesser degree. It is transported by selenoprotein P (Sepp1) in plasma and this carrier protein is a secreted glycoprotein [17]. However, the bioavailability of Se is reduced in the presence of heavy metals such as lead, which is highly toxic and is an inducer of OS [18]. In this sense, Se levels are decreased in patients with aortic abdominal aneurysm (AAA) in the aortic wall, and Se levels are inversely correlated with a high lead concentration [19]. This mechanism is poorly known, but a study has shown that Se levels are low in ruptured aneurysms [20].

Our results showed that Se concentrations were decreased in the TAA of MFS patients. Furthermore, there was a positive correlation with the activity of TrxR. This suggests that when Se concentrations decrease, the activity of this enzyme may diminish, while, when Se concentrations increase, its activity is restored. Se is a microelement or trace metal that promotes antioxidant activity through the 25 selenoenzymes. In the human body, Se concentration is inversely related to the risk of cardiovascular diseases [13]. In this regard, decreased plasma Se levels are associated with the development of different cardiovascular disorders, such as AAA in European populations [21]. The decrease in Se in the TAA of MFS patients may be due to: (1) Low dietary consumption of products with Se content by the MFS patients [17]. (2) Intestinal malabsorption syndrome in MFS patients [12,13,14]. (3) Different mutations present in selenoproteins, including the Sepp1 and the rs3877899G-rs7579G haplotype, which is present in the pathogenesis of AAA [22]. (4) Demand on the activity of selenoenzymes when trying to counteract the oxidant background present in TAA. (5) Deficiencies in vitamins A, D, and E. However, more studies are required to confirm these hypotheses.

In addition, mouse models of MFS show that Se deficit induces alterations in the wall of the arteries, which leads to endothelial dysfunction and hypertension and contributes to an OS background. Moreover, Se supplementation prevents the development of these alterations [20]. Taken together, our results, in addition to what has been reported in the literature on the state of Se levels, suggest that MFS patients could benefit from a Se-enriched diet or supplement.

The low Se levels in the TAA in MFS patients contribute to a significant decrease in the activities of TrxR and GPx. Regarding the activity of GPx, our results show a decrease in its activity in TAA of MFS patients, probably as a consequence of the decrease in Se and/or low availability of GSH, as has been reported in these patients [21,22,23,24]. In this sense, patients with Keshan disease who have dilated cardiomyopathy and Se deficiency present a reduced activity of GPx [25]. Furthermore, our research group has reported a decrease in the enzymes that employed GSH [9]. The GPx family is fundamental for the detoxification of organic peroxides and H_2_O_2_ due to the oxide-reduction process by the catalytic center of the selenocysteines under various physiological conditions, protecting cells against oxidative damage [26].

The TrxR/Trx system is also fundamental for the equilibrium of redox homeostasis. TrxR reduces thioredoxin (Trx) and is efficient in maintaining a reduced intracellular state. There are at least three TrxR isoforms in mammals—cytosolic, nuclear, and mitochondrial—and they play a role in the regulation of the inflammatory response and apoptosis [27]. This enzyme is elevated in the plasma of patients with heart failure and coronary atherosclerosis, associated with the presence of intraplaque hemorrhage [28]. High and low expressions of Trx in AAA have been observed in the luminal and abluminal faces of arteries, respectively [29]. However, other studies have reported a decrease in the activity of TrxR in pathologies with severe OS such as COVID-19 and metabolic syndrome [28,29,30]. TrxR also decreases in the presence of endothelial dysfunction and is associated with a prothrombotic and proinflammatory state [31]. In the presence of NADPH^+^, TrxR catalyzes the reduction of disulfide bridges between cysteines in proteins. It reduces the dysfunction of proteins, cellular receptors, and/or enzymes caused by ROS [32,33].

There are low levels of thiol proteins associated with a decrease in Se in plasma in patients with dissection of the TAA [34]. Lower thiol levels may be associated with a higher risk of developing TAA [35]. Our results show that total thiols are decreased and this was associated with the size of the area of lysis and the degree of overexpression of MMP9 and MMP2 in the TAA of the MFS patients. MMPs are produced by VSMC and by infiltration of monocytes and macrophages in the media or adventitia of the aorta in a pro-inflammatory state. They play an important role in the degradation of ECM in TAA from MFS patients, as evidenced by the increase in cystic necrosis. This result suggests that the loss of thiol bridges between the cysteines of the ECM proteins facilitates their degradation by MMPs, leading to ECM instability, and rendering it more susceptible to rupture. However, a study shows that treatment with TrxR in human N-type SK-N-SH cells may decrease the expressions of MMP9 and MMP2 [27,36].

The loss of thiol groups due to the loss of TrxR activity, which is associated with a decrease in Se concentration, contributes to the oxidative background present in MFS patients. This oxidative background can be reflected in the increased carbonylation in the TAA. In this sense, carbonylation is a marker of damage to amino acids such as lysine, methionine, and arginine, and also indicates the loss of disulfide bridges between the cysteines on the primary structure of the proteins by ROS [37]. At the same time, this contributes to endothelial dysfunction in the TAA.

Furthermore, the alteration of endothelial cells can contribute to the loss of redox homeostasis, where there is overproduction of O_2_^−^ and ONOO^−^ through the increased activities of the NADPH oxidases (NOX) including NOX1, NOX2, and NOS2 pathways, respectively. Our results show an increase in the expressions of NOX1 and NOS2, which contribute to the oxidative background and chronic inflammation in TAA in MFS patients. This result suggests an important role for these pathways in the development of the TAA in MFS patients. In this sense, an association between the serum concentration of pro-inflammatory cytokines and the diameter of the aneurysm has been demonstrated [38]. Our results show an increase in some pro-inflammatory markers such as toll-like receptor 4 (TLR4), IL-6, TNF, and TGF-β1. Regarding this, a positive correlation between TLR4, MMP9, and MMP2 and aneurysm development has been reported [39]. TLR4 is strongly associated with inflammation, and its signaling pathways play a key role in triggering the inflammatory process [39]. The overexpression of TNF-α and sICAM-1 is also associated with Se deficiency in AAA [20]. Our research group had previously demonstrated an increase in ICAM, TNF-α, TRVP-1, and NOS2 in the TAA in MFS patients [7].

**Figure 6:**
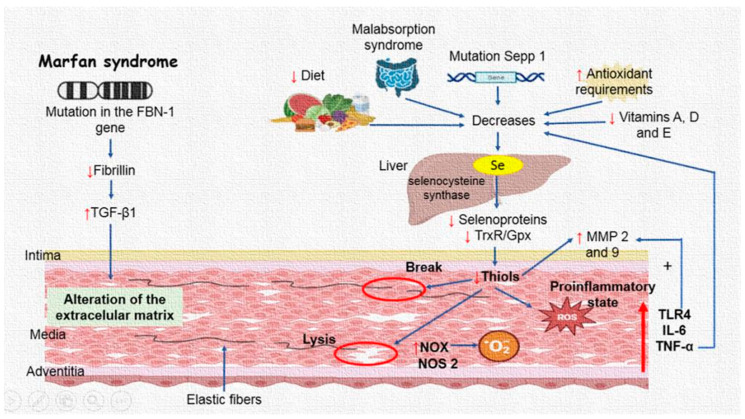
Selenium concentrations are decreased in the thoracic aorta aneurysm of Marfan syndrome patients, which can contribute to a decrease in TrxR and GPx activities and thiol groups. A decrease in the activities of these enzymes may contribute to the loss of redox homeostasis, which can, in turn, contribute to an increase in the pro-inflammatory interleukins associated with MMP9 and MMP2 overexpression. Abbreviations: Up arrow = increase, Down arrow = decrease, TGF-β1 = transforming growth factor beta-1, Se = selenium, MMP = metalloproteinases, TrxR = thioredoxin reductases, GPx = glutathione peroxidase, NOX = NADPH oxidases, TLR4 = toll-like receptor 4, NOS2 = inducible nitric synthase, IL = interleukins, TNF-α = tumoral necrosis factor.

The results of this study, in concert with those described in the literature, confirm that the aneurysm formation process in MFS patients is very complex with the participation of a chronic inflammatory process, an OS background, and anatomic and structural alteration of the aorta. All these alterations have their origin in the more than 800 mutations in the FBN1 gene and with TGF-β1 overexpression, which is involved in the development and maintenance of various tissues, including blood vessels and craniofacial growth [40]. The FBN1 mutations affect the intracellular kinase domain of fibrillin and alter TGF-β1 signaling, leading to the features present in MFS patients [41]. In addition, TGF-β1 can also regulate the expression of MMPs and elastase. The increase in the activity of elastase could enhance the degradation of EF associated with the reduction in the elasticity of the connective tissue that produces weakness in the aortic wall in the aneurysm [42]. These structural breaks in EF could be associated with decreases in GPx, TrxR, and inflammatory interleukins, and elevated ROS production that may affect the MMPs in the EMC [43]. Figure 6 summarizes the results presented in this article.

## 4. Materials and Methods

### 4.1. Ethical Considerations

This was an observational and comparative study that was performed in a prospective cohort of 19 patients versus 19 control subjects (CS) that attended the National Institute of Cardiology Ignacio Chávez and that were assisted in the aorta clinic of our institution. To classify a patient with MFS, more than two criteria are required, consisting of the following and which can be combined: family history, ectopia lentis, aortic dilatation, systemic score with >7/20 points, and genetic criteria with a demonstration of being positive for a mutation in the FBN1 gene [3]. The research protocol was approved by the research and ethics committee of the National Institute of Cardiology Ignacio Chávez (protocol number 23-1366). Each MFS patient signed an informed consent form in accordance with the Helsinki Declaration, as amended by the Congress of Tokyo, Japan [44].

### 4.2. MFS Patients and Control Subjects

Prior to surgery, an aortic tissue sample was obtained from MFS patients for analysis. Patients were considered candidates for surgery when they had ≥5 cm dilation and had been previously presented and discussed in a medical-surgical session or when they had attended the Institute for the first time with dilation and/or aortic dissection. Inclusion criteria were: Patients who met the Ghent criteria evaluated by a rheumatology expert and that required surgical intervention of the TAA, or another type of cardiovascular surgery, which was agreed upon under consensus in a medical-surgical session. The age of the patients was over 18 years old and they belonged to any gender. The exclusion criteria were: patients that did not agree to sign the informed consent form, MFS patients under 18 years of age; patients who did not suspend oral nitrates, NSAIDs, statins, calcium antagonist, or β-blocker intake 7 days prior to obtaining the samples; patients with neoplastic disease and/or associated infection; subjects with a smoking history in the last 4 years, patients with different phenotypic variants or phenotypes related to TAA, for example, Shiprintzen–Goldberg, Ehler–Danlos, Alagille, Weill–Marchesani, Turner, Noonan, Beals, MASS, and Loeys–Dietz (all its variants) syndromes, or with bicuspid aortic valve, autosomal dominant polycystic, kidney disease, pregnant women, and women with menopausal stage or having their menstrual period. A segment of the ascending TAA was taken during the surgery procedure of David type 5 or Bentall and De Bono, according to their arterial complication, and stored at 4 °C [45]. We revised the cabinet studies of the selected MSF patients in the clinical file. The studies had been requested during hospitalization to detect cardiovascular disorders and were recorded for further analysis. They included the determination of triglycerides, HDL, LDL, total cholesterol, creatinine, and glucose.

The tissue used for the CS was selected from patients that had an indication for surgery and in which aortic tissue could be obtained during the procedure that they required. The CS suffered from trivalvular aortic disease and underwent surgery for aortic stenosis. The surgery performed implied the substitution of aortic valves and the need to perform aortoplasty or resection of aortic tissue surrounding the valvular area. In addition, they did not have aortic damage and did not undergo surgery for aortic stenosis, having had no syndrome pathology diagnosed. In these patients, there was no suspicion of inflammatory disease or the presence of degenerative disorders such as thyroid and autoimmune diseases, arterial hypertension, and diabetes mellitus. Medications that could interfere with the outcome of the study such as lipid-lowering drugs or NSAIDs were suspended in the perioperative period. Cases were dealt with cautiously, to avoid including patients undertaking treatment with allopurinol, antioxidants, or probable inhibitors of ROS production. Warfarin, aspirin, clopidogrel, anticoagulant, antiplatelet medications, and other drugs were suspended.

### 4.3. Histology and Immunohistochemistry

A 2 mm segment from TAA tissue of the MFS patients (n = 19) and a segment of the thoracic aorta of the CS (n = 19) were washed in 0.9% NaCl for 30 s, three times. The solution was then decanted and phosphate buffer (pH 7.3) with 10% formalin was added for 24 h. The tissue of the aorta was cut to a thickness of 4 μm with a rotating microtome (Leica Biosystems, RM2125RT, Germany, Nussloch GmbH Heidelberger Str). The immunohistochemistry was processed according to the conventional histological technique. Briefly, 4 μm sections were mounted on slides treated with polylysine, and antigenic recovery with citrate buffer (0.1 M, pH 6.8) was made in a pressure cooker. The slides were mounted on cover plates and the technique was carried in the slide rack. This was incubated with the primary monoclonal antibodies (Marca Santa-Cruz) at a final dilution of 1:50 for all antibodies for TNF (c-4), sc-133192, IgG2a, the polyclonal antibody for NOS2 (c-19), sc-649, IgG, and the primary monoclonal antibodies (Abcam), anti-MMP9 antibody [EP1254] (ab76003) and anti-MMP2 antibody [EPR1184] (ab92536) for 2 h. Samples were then incubated for 30 min at room temperature with MACH2 Rabbit HRP-Polymer (Biocare Medical, Concord, CA, USA). The staining was paired for each antibody (CS and MFS groups) with their corresponding positive controls. The staining was revealed with DAB (3′3′-Diaminobenzidine), contrasted with hematoxylin, and mounted for observation and analysis. Histological sections were analyzed using a Carl Zeiss light microscope (63330 model) equipped with a Tucsen (9 megapixels) digital camera with TSview 7.1 software, at a 32× magnification. The intensity of light in the microscope was adjusted and remained constant. The photomicrographs were analyzed by densitometry using Sigma Scan Pro 5 Image Analysis software (Systat Software Inc. San Jose, CA, USA), and the parameters of analyses in the software were adjusted and remained constant for each of the antibodies. An average of five sections of endothelium and the muscular mean layer in each sample were examined. The density values are expressed as pixel units [7].

### 4.4. Thoracic Aortic Aneurysm Tissue Homogenization

Segments from the TAA were homogenized in liquid LN_2_, according to the methods that were previously described by Zúñiga-Muñoz et al. [9]. The Bradford method was utilized to determine the protein concentration in the homogenates. All assays of biochemical variables were made in duplicate.

### 4.5. TrxR and GPx Activities

For the determination of TrxR and GPx activities, 100 μg of the protein of the TAA homogenate was utilized according to previously described methods [9,23]. GPx activity is expressed as nmol of NADPH^+^ oxidized/min/mg protein with an extinction coefficient of 6220 M^−1^ cm^−1^ at 340 nm for NADPH^+^. TrxR activity was determined indirectly by the amount of DTNB in the presence of NADPH^+^ to form 2 moles of TNB. DTNB oxidation was monitored at 412 nm at 37 °C for 6 min with an extinction coefficient of 13,600 M^−1^ cm^−1^. A total of 100 μg of TAA homogenate protein suspended in 3 mL of 0.1 mM phosphate buffer (KH_2_PO_4_, pH 7.0) was added to 0.2 mM NADPH^+^, 1 mM EDTA, and 0.1 mg/mL BSA. The samples were read in the presence of 20 μL of the specific TrxR inhibitor (10 μM auranofin) and together with a duplicate of the sample without the inhibitor.

### 4.6. Oxidative Stress Markers

For the determinations of the OS markers, 100 µg of protein from the TAA homogenate was used, except for the Se and carbonylation determinations, where 200 µg was used. Carbonylation was processed according to the method described by Zúñiga-Muñoz et al. [9]. The absorbance was read at 370 nm, using air as the blank and a molar absorption coefficient of 22,000 M^−1^ cm^−1^. Se determination in the TAA homogenate was performed according to the method described by Soto et al., and the absorbance was read at 600 nm [23]. The determination of total thiol groups was performed as previously described by Erel and Neselioglu [34]. The absorbance was measured at 415 nm.

### 4.7. Determinations of IL-1β, TNF, IL-6 and TGF-β1

Determination of IL-6 and TGF-β1 in TAA was made with ELISA kits provided by Abcam, Human IL-6 (ELISA Kit. Cat ab178013) and Human TGF-β1 (ELISA Kit Cat ab100647) and were measured at a wavelength of 450–492 nm using a visible light microplate reader (Stat Fax 3200 Awareness Technology, Palm City, FL, USA) according to the manufacturer’s specifications.

### 4.8. Determinations of NOX1 and NOS2 by Western Blotting

A total of 50 μg of protein of the TAA homogenate was run on 10% SDS-PAGE, blotted onto a polyvinylidene difluoride membrane (0.22 μm Millipore, Billerica, MA, USA), and then blocked for 1 h at room temperature with Tris-buffer solution −0.01% Tween (TBS-T 0.01%) plus 5% non-fat milk. The membranes were incubated overnight at 4 °C with mouse primary polyclonal antibodies, Anti-NOX1 antibody (ab55831), and Anti-TLR4 antibody [7E3] (ab105950) provided by Abcam (Cambridge, CB2 0AX, UK) at a final dilution of 1:1000. Finally, the membranes were incubated overnight at 4 °C with a secondary antibody that was conjugated with horseradish peroxidase at a dilution of 1:10,000 (Santa Cruz Biotechnology, Santa Cruz, CA, USA). All of the blots were incubated with α-actin antibody as load control.

### 4.9. Statistical Analysis

Sigma Plot version 14.5 (SigmaPlot^®^ version 14.5, Systat Software Inc. 2107, San Jose, CA95131 EE.UU, North First Street, Suite 360, Jandel Corporation, San Jose, CA, USA) was used for statistical analysis and graph plotting. The data are presented as median and minimum and maximum range. Statistical significance was determined by the Mann–Whitney U rank sum test followed by the normality test (Shapiro–Wilk) Differences were considered statistically significant when *p* ≤ 0.05.

## 5. Conclusions

Se concentrations are decreased in the TAA of MFS patients. This can contribute to a decrease in TrxR and GPx activities and thiol groups. A decrease in the activities of these enzymes may contribute to the loss of redox homeostasis, which can, in turn, contribute to an increase in the pro-inflammatory interleukins associated with MMP9 and MMP2 overexpression.

### Study Limitations

The major limitation of our study is the relatively small study population for both the MFS patients and CS. The obtaining of tissue from aortic samples is very difficult despite the informed consent and the aortic sample size is very small. In addition, the results from this study suggest the importance of giving a selenium-enriched food supplement to these patients, which could probably contribute to improving their quality of life. Another limitation is the improbability of having matched controls for age and gender since it is not possible to obtain tissue samples from healthy people. Tissue samples can only be obtained from subjects having a surgical indication where there is a possibility to ethically withdraw a small sample. This depends on the surgical technique used and the informed consent of the patients. However, even though there are age differences in the CS and MFS patients, we can be certain that there were no comorbidities or aortic damage as shown by the imaging studies reported.

## Figures and Tables

**Table 1:** Patients classified as MFS who met the Ghent criteria.

Patients	Age	Sex	HFH	AD	EL	SS	Total Systemic Score	Gen FBN1	Total Ghent Criteria
1	38	M		+		+	10	+	3
2	29	M	+	+	+	+	14		4
3	33	M	+	+	+	+	16		4
4	56	F		+		+	12		2
5	20	M		+	+	+	18		3
6	25	F		+		+	7		2
7	27	F		+	+	+	8	+	4
8	36	F	+	+		+	12		3
9	41	F	+	+		+	12		3
10	37	F	+	+		+	12		3
11	27	M	+	+		+	9		3
12	51	M		+		+	12	+	3
13	61	F	+	+		+	13	+	4
14	29	F	+	+	+	+	12		4
15	17	M		+	+	+	12		3
16	31	M		+	+	+	14		3
17	60	M	+	+	+	+	11		4
18	45	M		+		+	10	+	3
19	43	M	+	+		+	7		3

Abbreviation: M = male, F = female, HFH = hereditary family history, SS = systemic score (subjects who met more than 7/20 of the points necessary to classify within this criterion), AD = aortic dilatation, EL = ectopia lentis, G-FBN1 = FBN1 gene mutation.

**Table 2:** Demographic variables and serum biochemistry in MFS patients and CS.

	Total n = 38 (100%)	MFS n = 19 (50%)	CS n = 19 (50%)	*p*
Demographic variables in mean ± SE				
Age	39 ± 15	35 ± 12	43 ± 17	NS
Weight	75 ± 17	75 ± 13	75 ± 20	NS
Size	1.70 ± 0.13	1.77 ± 0.09	1.63 ± 0.11	0.0001
BMI	25.6 ± 5.1	23.7 ± 3.4	27.5 ± 5.9	0.01
Cholesterol	150.2 ± 48.9	148.7 ± 45.2	151.7 ± 53.3	NS
C-HDL	39.2 ± 11.6	37.3 ± 13.2	41.2 ± 9.6	NS
C-LDL	97.7 ± 33.3	97.7 ± 30.5	97.8 ± 43.8	NS
Triglycerides	146 ± 82	146 ± 79	145 ± 86	NS
Glucose	102 ± 26	100 ± 28	104 ± 25	NS
Creatinine	0.88 ± 0.44	0.93 ± 0.56	0.83 ± 0.29	NS
Comorbidities (%)				
Diabetes Mellitus	3 (7)	0	3 (13)	0.07
SAH	16 (33)	5 (21)	11 (46)	0.06
Hypothyroidism	1 (2)	0	1 (4)	NS
Alcoholism	13 (27)	7 (29)	6 (25)	NS
Smoking	19 (40)	10 (42)	9 (38)	NS
Echocardiography evaluation (% or median with the Min–Max or mean ± SE)				
LVEF	54 ± 11	50 ± 11	57 ± 11	0.02
Surgical				
Aneurysm	22 (49)	11 (46)	21 (88)	0.002
Dissection	16 (67)	13 (54)	3 (13)	0.002
Clamp-Time	143 (46–253)	154 (46–253)	128 (48–250)	NS
Extracorporeal circulation	187 (93–440)	197 (148–336)	177 (93–440)	NS
EURO-SCOREII	3 (4–17)	3 (4–12)	2 (5–17)	NS

Abbreviation: BMI = body mass index, C-HDL = cholesterol high-density lipoprotein, C-LDL = cholesterol low-density lipoprotein, LVEF = left ventricular ejection fraction, EURO-SCOREII = European system for cardiac surgical risk assessment, SAH = systemic arterial hypertension, MFS = Marfan Syndrome, CS = control subjects, NS = not significant.

**Table 3:** Interleukins, Se, and carbonylation concentrations in TAA in MFS patients and in a segment of the thoracic aorta from control subjects.

Variables (mg of Protein)	CS Median (Min–Max)	MFS Median (Min–Max)
IL-6 (pg)	227.1 (0.0–888.96	409.1 (111.3–1137.1) ***
TGF-β1 (pg)	570.3 (21.7–1076.3)	964.6 (384.2–3086.6) **
Carbonylation (nmol carbonyls)	2.3 (0.8–3.6)	2.5 (1.7–7.5) †
Se (pg)	224.2 (183.2–322.4)	205.5 (24.4–243.9) *
Thiols (µM)	196.4 (119.1–286.5)	164.8 (58.2–246.7) *

*** *p* = 0.004, ** *p* = 0.04, * *p* = 0.03, † *p* = 0.02

## Data Availability

The datasets generated and analyzed during the current study are available from the corresponding author upon reasonable request.
